# Knowledge, beliefs and attitudes of physicians in low and middle-income countries regarding interacting with pharmaceutical companies: a systematic review

**DOI:** 10.1186/s12913-016-1299-4

**Published:** 2016-02-17

**Authors:** Tamara Lotfi, Rami Z. Morsi, Mhd Hashem Rajabbik, Lina Alkhaled, Lara Kahale, Hala Nass, Hneine Brax, Racha Fadlallah, Elie A. Akl

**Affiliations:** Department of Clinical Research Institute, American University of Beirut, Beirut, Lebanon; Department of Pediatrics and Adolescent Medicine, Faculty of Medicine, American University of Beirut, Beirut, Lebanon; Faculty of Health Sciences, American University of Beirut, Beirut, Lebanon; Faculty of Medicine, University of Damascus, Damascus, Syria; Faculty of Medicine, Université Saint Joseph, Beirut, Lebanon; Department of Internal Medicine, American University of Beirut, Beirut, Lebanon; Department of Clinical Epidemiology and Biostatistics, McMaster University, Hamilton, ON Canada; Department of Internal Medicine, AUBMC, Riad-El-Solh Beirut, 1107 2020 P.O. Box: 11–0236, Beirut, Lebanon

**Keywords:** Knowledge, Beliefs, Attitudes, Physicians, Pharmaceutical company representatives

## Abstract

**Background:**

Understanding the perceptions and attitudes of physicians is important. This knowledge assists in the efforts to reduce the impact of their interactions with the pharmaceutical industry on clinical practice. It appears that most studies on such perceptions and attitudes have been conducted in high-income countries. The objective was to systematically review the knowledge, beliefs and attitudes of physicians in low and middle-income countries regarding interactions with pharmaceutical companies.

**Methods:**

Eligible studies addressed any type of interaction between physicians and pharmaceutical companies. The outcomes of interest included knowledge, beliefs and attitudes of practicing physicians. The search strategy covered MEDLINE and EMBASE databases. Two reviewers completed in duplicate and independently study selection, data abstraction, and assessment of methodological features. The data synthesis consisted of a narrative summary of the findings stratified by knowledge, beliefs and attitudes.

**Results:**

We included ten reports from nine eligible studies, each of which had a number of methodological limitations. Four studies found that the top perceived benefits of this interaction were receiving information and rewards. In five out of eight studies assessing the perception regarding the impact of the interaction on the behavior of physician prescription, the majority of participants believed it to be minor. In one of these studies, participants perceived that impact to be lesser when asked about their own behavior. The attitudes of physicians towards information and rewards provided by pharmaceutical company representatives (PCRs) (assessed in 5 and 2 studies respectively) varied across studies. In the only study assessing their attitudes towards pharmaceutical-sponsored Continuing Medical Education, physicians considered local conferences to have higher impact. Their attitudes towards developing policies restricting physicians’ interactions with PCRs were positive in two studies. In one study, the majority of participants did not mind the public knowing that physicians were receiving gifts and awards from drug companies.

**Conclusions:**

This review identified few studies conducted in low and middle-income countries. While physicians generally perceived the impact of interactions on their behavior to be minor, their attitudes toward receiving information and rewards varied across studies.

**Electronic supplementary material:**

The online version of this article (doi:10.1186/s12913-016-1299-4) contains supplementary material, which is available to authorized users.

## Background

The appropriateness of marketing relationships between physicians and the pharmaceutical industry has been debated since the 1960s [1]. In 2012, the total expenditure on drug promotion exceeded $28 billion in the US alone, $20 billion in five European countries (UK, France, Germany, Spain, Italy), and $26 billion in Japan [2].

Campbell et al. found that 84 % of doctors in the United States reported some form of relationship with the pharmaceutical or medical device industries in 2009 [3]. Also, pharmaceutical and medical device industries found up to 60 % of accredited continuing medical education costs in the US [4]. These types of interactions appear to be more prevalent in certain low and middle-income countries. A 2012 study in Libya reported that 94 % physicians in public and private practice received at least one visit in the preceding year [5]. Another study in Izmir Turkey found that pharmaceutical company representatives visited 90 % of physicians at least once per week [6].

A number of studies have found that pharmaceutical drug promotions can influence demand for prescription drugs [7], and physician visits for conditions treated by heavily advertised drugs [8]. More specifically, there is evidence of an association between exposure to the information provided by pharmaceutical company representatives (PCRs) and a greater frequency of prescribing [9].

In spite of the above evidence, most doctors believe that that their interaction with the pharmaceutical industry does not influence their prescription behavior [10]. A number of studies found that, while doctors may acknowledge that such interaction may influence others, they believe it does not influence them personally [11, 12].

Physician’s perceptions and attitudes might hinder efforts to reduce the impact of the pharmaceutical industry on clinical practice. As a result, it would be important to synthesize the evidence on the knowledge, beliefs and attitudes of physicians. There are a number of factors related to the level of country income that might affect the perceptions and attitudes of physicians. One of those factors is the existence of tighter regulatory control in high-income countries. For example, in the States, the Sunshine act requires drug and device manufacturers to report payments and items of value given to physicians. The existence of such regulations may increase physicians’ awareness but also affect their attitude towards accepting payments and items of value. Also, it appears that most studies of physicians’ perceptions and attitudes have been conducted in high-income countries. Therefore, our objective was to systematically review the knowledge, beliefs and attitudes of physicians in low and middle-income countries regarding interactions with pharmaceutical companies.

## Methods

### Eligibility criteria

The inclusion criteria were:

- Type of study design: quantitative design (e.g., survey study) and qualitative design (e.g., focus group, interviews, semi-structured interviews);
- Types of participants: physicians practicing in low or middle-income countries (LMIC). We used the World Bank income classification of countries’ income level;
- Types of interactions: any form of interaction between physicians and pharmaceutical companies or PCRs (e.g., gifts, meeting with representatives of drug companies or medical/surgical device manufacturers; receiving free drug samples, industry-provided meals; pharmaceutical-funded research; pharmaceutical-sponsored continuous medical education including travel funding; consultancy; stock ownership);
- Types of outcomes: for the purpose of this study, we used the following classification [13]:

- ◦ Knowledge (e.g., related to the extent of the interaction between physicians and pharmaceutical companies);
- ◦ Beliefs: (e.g., perceptions of the effect of the interaction on quality of patient care);
- ◦ Attitude: (e.g., toward the appropriateness and acceptability of the interaction).

We excluded studies that focus only on residents, patients or general public and studies not published in English.

### Search strategy

A medical librarian assisted with designing the search strategy (Additional file 1). We electronically searched in September 2015 the MEDLINE and EMBASE databases using the OVID interface. The search strategy combined terms for ‘physicians’ and ‘pharmaceuticals’, and used free text words and medical subject heading. No search filter was used. In addition, we reviewed the reference lists of included studies and searched the grey literature (theses and dissertations).

### Selection of studies

Teams of two reviewers screened in duplicate and independently titles and abstracts of identified citations for potential eligibility. We obtained the full texts of citations judged as potentially eligible by at least one of the two reviewers. Then, the teams of two reviewers screened in duplicate and independently the full texts for eligibility. The reviewers resolved disagreements by discussion or with the help of a third reviewer. We conducted calibration exercises and used a standardized and pilot tested screening form. We calculated the kappa statistic of agreement between the reviewers.

### Data collection

Teams of two reviewers used a standardized and pilot tested screening form with detailed written instructions to abstract data from eligible studies. Disagreements were resolved by discussion or with the help of a third reviewer. Abstracted data included the: study design, funding source, characteristics and setting of the participants, type of interaction addressed, and results.

### Assessment of methodological features of included studies

Teams of two reviewers assessed in duplicate the methodological features of each eligible study. They resolved disagreements by discussion or with the help of a third reviewer. The Criteria assessed were: sample size calculation, reporting of a sampling frame, the sampling method, the response rate, and the validity of tool.

### Data analysis and synthesis

We assessed the agreement between reviewers for full text screening by calculating the kappa statistic. We did not conduct a meta-analysis due to the nature of the data. Instead, we narratively summarized findings stratified by knowledge, beliefs and attitudes.

## Results

### Results of the search

**Fig. 1:**
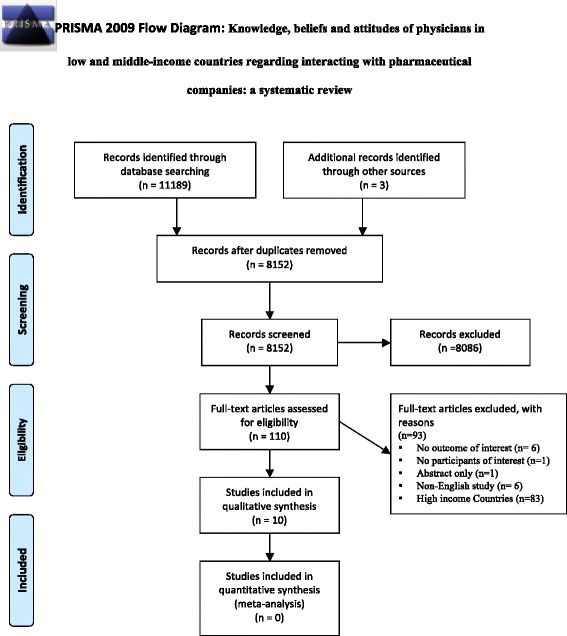
The study flow

Figure 1 shows the study flow. Of 11,189 citations captured by the search strategy, we identified ten reports of nine eligible studies. One of the studies described both quantitative and qualitative data in two separate reports [14, 15]. The kappa statistic for full-text screening was high at 0.893.

Table 1 describes the characteristics of the participants, setting, and type of interaction addressed in each of these studies. The studies were conducted in six different countries: Yemen [14, 15], Libya [16], Turkey, Nigeria [17], India [18] and Pakistan [19], Malaysia [20], Iraq [21] and Brazil [22].

**Table 1:** Characteristics and methodological features of the included studies

Study ID Study design	Participants and setting	Type of interaction studied	Sampling and response rate	Validity of tool; pilot testing	Results
Al Areefi 2013 Study 1 [15] • Semi-structured interview Study 2 [14] • In-depth interview	Study 1 & 2 • Physicians from private and public hospitals (*N* = 32) • Sana’a, Yemen • May-July 2009	Study 1 • Relationship with PCR • Frequency of PCR visits • PCR marketing activity Study 2 • Interaction between PCRs and physicians • Physicians’ attitudes toward these interactions and the PCRs • Reasons for accepting the PCRs’ visits	• Sample size calculation (both studies): not reported • Sampling frame (both studies): bot reported • Sampling method (both studies): purposeful sampling • Response rate (both studies): 100 %	Both studies • Interview guide developed through a literature review, then pilot tested with 3 physicians	Study 1 • Percentage of participants reporting the following as important factors in prescribing a specific drug: relationship with PCR (9 %), frequency of PCR visits (34 %), PCR marketing activity (13 %). Study 2 • Most physicians thought they were immune from being influenced by their interactions with PCRs. • Physicians accept a PCR’s visit because of the beneficial patronage or the financial support provided by the PCRs. • Physicians recognize the professional authority of PCRs as information providers. • Physicians considered accepting the PCR’s visit was their moral duty. • Participants still doubt the role of representatives, with some accusing them of creating problems, harming the ethical reputation of the profession and harming the patients’ welfare
Alssageer 2013 [16] • Self-administered anonymous questionnaire	• Doctors from selected public and private practice settings (*N* = 608) • Libya • August-October 2010	• Gifts received from PCRs (e.g., printed materials, simple gifts or drug samples	• Sample size calculation: not reported • Sampling frame: not reported • Sampling method: convenience sampling • Response rate: 61 %	• Questionnaire developed based on previous published studies	• Perceived benefits from interactions with PCRs: receiving new information about products (95 % approved), invitations to conferences (35 % approved) and receipt of gifts (22 % approved). Attitudes towards accepting PCR gifts: 25 % totally disapproved; 25 % clearly approved; 50 % would accept gifts in some cases. • Acceptance of gifts according to educational value: of respondents who did not disapprove of gift provision, 82 % considered educational gifts as appropriate; 49 % considered non-educational gifts as appropriate. Belief that pharmaceutical promotional activity decreased rational drug prescribing: 42 % disagreed; 32 % agreed; and 27 % were neutral. • Perceived impact of pharmaceutical promotion on prescribing decisions of physicians: minor (62 %); major (38 %). Perceived impact of pharmaceutical promotion on own prescribing decisions: minor (80 %); major (20 %). Perceived need to develop national policies to restrict PCR interactions with doctors: 57 %. • Awareness of guidelines regarding PCR interaction: 99 % had never read any guidelines.
Guldal 2007 • Interview	• Specialists and general practitioners in government posts (*N* = 446: 24 % GPs, 42 % specialists and 35 % residents) • Turkey	• Visits by PCR (frequency, duration) • Promotional Program • Provision of drug information	• Sample size calculation: not reported • Sampling frame: list of physicians from the Ministry of Health and from the 1992 Izmir telephone directory • Sampling method: stratified random sampling • Response rate: 91 %	• Questionnaire pretested with 25 subjects	• Physicians’ expectations about promotional programs: reliable educational publications (82 %); medical equipment (57 %); free drug samples (54 %); financial support for training courses (43 %); social events (e.g., dinners, trips) (34 %); and gifts of up to $50 for private use (27 %). • Support for the prohibition of PCR visits to physicians: 54 % • Attitudes towards promotional programs: not ethical (33 %); not ethical in some aspects (36 %); ethical (20 %). • Perceived effect of advertising gifts on prescriptions: high (18 %); medium (12 %); low (44 %); no effect (27 %) • 68 % thought the information was unreliable. • 94 % pointed out the necessity for a reliable source of information other than drug companies. • 54 % approved that doctors who receive expensive advertising gifts tend to prescribe that company’s products • Public knowledge that physicians were receiving gifts and awards from drug companies did not matter for 64 %
Loh 2007 [20] • Self-administered questionnaire	• Registered practitioners (*N* = 172) • Penang, Malaysia • March to May 2005	• Pharmaceutical- sponsored continuous medical education (CME)	• Sample size calculation: not reported • Sampling frame: databases of Penang Medical Practitioners’ Society and Malaysian Medical Association (Penang branch) • Sampling method: exhaustive (all registered doctors) • Response rate: 19.5 %	• Self-developed tool: content first approved by the Committee of The Penang Medical Practitioners’ Society, reviewed by 5 clinicians in active medical service to ensure clarity and appropriateness • Pilot testing not reported	• Rated impact on clinical practice by descending order, as it relates to medical conferences: local conferences, pharmaceutical talks, internet-based medical education, conferences organized by pharmaceutical firms and overseas conferences • Rated impact on clinical practice by descending order, as it relates to pharmaceutical firms: reputation of the firm, pharmaceutical company representatives, and advertisement or announcement.
Mikhael 2014 [21] • Self-administered questionnaire	• Specialist physicians in different areas of Baghdad governorate (*N* = 22) • Iraq • March to October 2013	• Quality of promotional information that is given by MRs to physicians	• Sample size calculation: not reported • Sampling frame: not reported • Sampling method: not reported • Response rate: 63 %	• Self-developed tool; validation not reported. Pilot testing not reported	• Information from PCRs about drug indication was perceived as good and information about drug contraindications and side effects was perceived as weak. • Academic physicians have a significantly more negative opinion than hospital physicians regarding PCRs information on drug contraindication • Only hospital physicians found that PCRs’ information are useful for them
Oshikoya 2011 [17] • Self-administered questionnaire	• Doctors in University College Hospital teaching hospital (*N* = 163) • Nigeria	• Provision of drug information	• Sample size calculation: not reported • Sampling frame: not reported • Sampling method: convenience sampling • Response rate: 41 %	• Questionnaire developed from previous studies in developed and developing countries, then piloted among 10 doctors	• Drug information was sourced from colleagues (99 %), drug reference books (97 %), PCRs (93 %), materials from drug companies (93 %), scientific papers/journals/internet (91 %), and drug promotion forum/product launches (88 %). • Perception of importance of PCR as drug information source: efficient (70 %), reliable and accurate (66 %), influences prescription behavior (72 %), useful and readily used when prescribing (69 %) • Perception of the effect of detailing by a PCR of a promoted drug: increases awareness (82 %), increases preference for prescription (60 %).
Rajan 2008 [18] • Self administered survey questionnaire	• General practitioners and specialists from an urban town (*N* = 57) • India	• Provision of drug information	• Sample size calculation: not reported • Sampling frame: not reported • Sampling method: convenience sampling • Response rate: 95 %	• Questionnaire based on theoretical model, no validation reported	• Perception that product information provided by medical representatives is biased and insufficient: 79 %
Scheffer 2014 [22] • Structured Interview	• Physicians in Sao Paolo, Brazil (*N* = 300) • October 2007 to May 2009	• Informative materials about ARVs • Visits by sales promoters and sales representatives • Inexpensive objects for the doctor’s office • Invitations to take part in continuing education courses and events Scientific journals sponsored by the laboratories	• Sample size calculation: described in detail • Sampling frame: Logistics Control System (SICLOM) of the STD, AIDS and Viral Hepatitis Department of the Ministry of Health in Sao Paolo • Sampling method: stratified random sampling • Response rate: not reported	• Validation not reported; pilot testing not reported	• Pharmaceutical companies’ actions were considered to have a strong influence (10 %), slight influence (50 %) or no influence (40 %) on physicians’ prescribing of antiretroviral.
Siddiqi 2011 [19] • Self administered questionnaire	• General practitioners and consultants (*N* = 200) • Various districts of Rawalpindi division, Pakistan • January –June 2010	• Sponsorships • Scientific promotional tools • Personal touch promotional tools Common promotional tools.	• Sample size calculation: not reported • Sampling frame: not reported • Sampling method: “Judgmental sampling” • Response rate: 75 %	• Questionnaire was adapted from existing one	• General practitioners perceived common promotional gifts as most effective tool for changing the prescribing behavior; while sponsorship and personal touch promotional tools are considered neutral and relatively least important. • Consultants perceived scientific promotional tools as most influencing in changing prescribing behaviors in comparison with other promotional tools; while sponsorships are least important

The specialties of physicians included were: both general practitioners and specialists in five studies [18–20, 22]; specialists only in one study [21] physicians from private and public hospitals in three studies [14–16, 20]; and not specified in one study [17]. The types of interaction assessed were: “visits by PCRs” in three studies [14, 15, 22]; “PCR marketing activity” (promotional tools such as gifts or sponsorships) in four studies [16, 19, 22]; “PCR as source of drug information” in four studies [14, 15, 17, 18, 21] and pharmaceutical-sponsored continual medical education (CME) [20] and invitations to take part in CME courses [22].

### Methodological features

Table 1 also describes the methodological features of the included studies. There were a number of methodological limitations: only one study reported sample size calculation [22]; one study used random approach to sampling and another used stratified random sampling [22] and both described their sampling frame [22]; and response rates across studies varied between 19.5 and 100 %.

### Findings

Table 1 describes the findings of each study. The findings addressed beliefs (measured as perceptions) and attitudes but did not address knowledge. Below, we narratively summarize these findings organized by the following topics:

1. Perceived benefits of the interaction (*n* = 4 studies);
2. Perceived impact of the interaction (*n* = 8 studies);
3. Attitudes towards information provided by PCRs (*n* = 5 studies);
4. Attitudes towards rewards provided by PCRs (*n* = 2 studies);
5. Attitudes towards policies that regulate the interaction (*n* = 2 studies);
6. Attitudes towards public knowledge of the interaction (*n* = 1 study);
7. Attitudes towards pharmaceutical-sponsored CME (*n* = 1 study)

1. Perceived benefits of the interaction (*n* = 4 studies):
  In one study, the percentages of participants who agreed with the following as benefits from interactions with PCRs were receiving: new information about products (95 %), invitations to conferences (35 %) and gifts (22 %) [16].
  In another study, physicians’ expectations about promotional programs from drug companies included: reliable educational publications (82 %); medical equipment (57 %); free drug samples (54 %); financial support for training courses (43 %); social events (e.g., dinners, trips) (34 %); and gifts up to $50 for private use (27 %). In a third study, the majority of participants (82 %) were in favor of the statement “detailing of a PCR increases my awareness” [17]. In the study reporting qualitative data, physicians considered the medical representatives as “information providers”. They also reported “beneficial patronage” and “financial support” as reasons to accept their visits [14].
2. Perceived impact of the relationship (*n* = 8 studies):
  In one of the studies, the effect of gifts on prescriptions was perceived as high (18 %), medium (12 %), low (44 %); and no effect (27 %). In the same study, 54 % of the participants approved that doctors who receive expensive advertising gifts tend to prescribe that company’s products. Another study found that the effect of the promotional tools of PCRs on the prescribing practices of physicians in general was perceived as minor by 62 % of the participants and major by 38 %. Its impact on one’s own prescribing practices (as opposed to ‘in general’) was perceived as minor by 80 % and major by 20 % [16]. In the same study, 42 % disagreed that pharmaceutical promotional activity decreased rational drug prescribing, 32 % agreed, and 27 % were neutral [16].
  In the study reporting qualitative data, participants accused the PCRs of creating problems, harming the ethical reputation of the profession and, harming the patients’ welfare. At the same time, most physicians thought they were “immune” from being influenced by their interactions with PCRs [14].
  Another study found that the majority of its participants (60 %) were in favor of the statement “detailing of a PCR increases my preference for prescribing the promoted drug” [17].
  The study conducted in Pakistan compared general practitioners and consultants regarding their perceptions of the sponsorships and three types of promotional tools: “scientific”, “personal touch”, and “common” (no further details were given). The tool considered as the most effective for changing prescribing behavior was “common promotional gifts” for general practitioners, and “scientific promotional tools” for consultants [19]. In another study, the participants reported the following as important factors in prescribing a specific drug: relationship with PCR (9 %), frequency of PCR visits (34 %), and PCR marketing activity (13 %) [15].
  In a study assessing the impacts of CME on clinical practice, physicians rated the reputation of the firm as highest impact, followed by pharmaceutical company representatives and advertisement or sponsored announcement as lowest [20].
  One study asked the physicians to rate the influence of all actions of pharmaceutical companies on their prescribing behavior and the majority considered it of slight influence (50 %) or no influence (40 %) [22].
3. Attitude towards information provided by PCRs (*n* = 5 studies):
  Five studies assessed the attitudes of physicians towards the information provided by PCR. In the first study, the importance of PCR as drug information source differed between the participants: efficient (70 %), reliable and accurate (66 %), influences prescription behavior (72 %), and useful and readily used when prescribing (69 %) [17]. In the second study, 68 % of the participants thought that the information was unreliable, and 94 % pointed out the necessity for a reliable source of information other than drug companies. In the third study, product information provided by medical representatives was perceived as biased and insufficient (79 %) [18]. In the study reporting qualitative data, physicians recognized the professional authority of PCRs as information providers [14]. In the fifth study, physicians in Iraq considered that information provided by PCRs concerning drug indication was good and that concerning drug contraindication and side effects was weak [21]. In this study, hospital physicians found that information provided by PCRs were useful while academic physicians did not [21]. The difference between opinions of academic and hospital physicians was significant concerning the focus of PCRs on cost difference in their promoted product [21].
4. Attitude towards rewards provided by PCRs (*n* = 2 studies):
  One study found that 25 % of participants totally disapproved of receiving gifts, 25 % clearly approved, and 50 % would accept gifts in some cases. Of those who did not disapprove of gifts, 82 % considered educational gifts appropriate and 49 % considered non-educational gifts as appropriate [16]. One study asked participants about how ethical it is to receive promotional programs: 33 % found it ‘not ethical’, 36 % found it ‘not ethical in some aspects’, and 20 % found it ‘ethical’.
5. Attitudes towards policies that regulate the interaction (*n* = 2 studies):
  Two studies discussed the perceptions and attitudes towards policies that restrict the interactions of physicians with PCRs. In the first study, 54 % of the participants were in support of the restrictions. In the second study, 57 % approved of developing such policies [16]. That same study found that 99 % of the participants had never read any guidelines regarding PCR interactions [16].
6. Attitudes towards public knowledge of the interaction (*n* = 1 study):
  One study found that for 64 % of the participants, public knowledge that physicians were receiving gifts and awards from drug companies did not matter and 25 % reported they would mind and try to hide it.
7. Attitudes towards pharmaceutical-sponsored CME (*n* = 1 study)
  One study assessed the attitude of general practitioners and specialists towards the pharmaceutical-sponsored CME. This study found that local conferences were considered to have a higher impact on clinical practices than pharmaceutical talks, internet-based medical education, conferences organized by pharmaceutical firms and overseas conferences [20].

## Discussion

### Summary of findings

We identified nine studies assessing the knowledge, beliefs and attitudes of physicians regarding interactions with pharmaceutical companies, in low and middle-income countries. The top reported perceived benefits of the interaction related to receiving new and reliable information. Participants perceived that the impact of the relationship on physicians’ prescription behavior was minor. They perceived it to be lesser when asked about their own behavior. Physicians’ attitudes towards information and towards rewards provided by PCRs varied across studies. Their attitudes towards developing policies restricting the interaction of physicians with PCRs were positive. In one study, the majority of participants did not mind the public knowing that physicians were receiving gifts and awards from drug companies.

### Strengths and limitations of the review

This is the first published systematic review about the knowledge, beliefs and attitudes towards the interaction of physicians in low and middle-income countries with PCRs. Also, the last published systematic review on this topic in high-income countries that we are aware of was published in 2000 [10]. One limitation is that we only included studies published in English. Other limitations relate to shortcomings of the available primary studies: none assessed knowledge, they used different, and mostly non-validated questionnaires, and were conducted in different settings with varying cultural and social backgrounds.

### Comparison to findings of similar reviews

We did not find other similar reviews conducted among physicians in the region of our interest. A recently published systematic review focusing on medical students, from high-income countries and one middle-income country, found variable attitudes towards pharmaceutical marketing practices. The attitudes of students were generally not in favor of restricting visits from PCRs or sponsored educational presentations [23]. The older systematic review published in 2000 conducted on both physicians and residents in high income countries found positive attitudes towards the information from PCRs and that physicians approved that the interaction affects their prescription behavior, more than residents did [10] while our review found mixed attitudes. In the absence of studies directly comparing attitude of physicians in low and middle-income countries to those in high-income countries, it is hard to infer how they compare.

Our findings suggest that, while physicians are aware of the potential influence of the interaction with pharmaceutical companies, they believe that they are themselves less prone to that influence. A recently published study found that the majority of doctors in Germany believed that their prescribing habits were not influenced by PCR visits [24]. Another study conducted in Argentina found that half of the doctors believe that the benefits from pharmaceutical industry influence medical prescription. However, only 27 % believed that such benefits influence their own prescriptions [25]. Such beliefs, in addition to the perceived benefits of the interaction, likely contribute to some of the positive attitudes towards information and rewards provided by PCRs.

### Implications for policy makers

There is a need for policy interventions related to interactions between physicians and PCRs to maximize their potential benefits (e.g., receiving drug related information) and minimize their potential harms (e.g. negative impact on physicians’ prescription behavior) [26]. In order for these interventions to be successful, they need to take into account the knowledge, beliefs and attitudes of physicians. Based on the findings of this review, such interventions need to raise awareness amongst physicians about the evidence that pharmaceutical drug promotion does affect prescription behavior. The fact that the majority of physicians are in favor for developing policies that restrict physicians’ interactions with PCRs suggests that such policies would be acceptable and likely effective. As noted earlier, such policies might be more challenging to introduce and implement in low and middle-income countries compared to high-income countries where these are already being introduced (e.g. Sunshine act in the USA).

### Implications for future research

Our findings show a significant gap in the published research concerning our topic, particularly in low and middle-income countries. Future studies should attempt to directly compare the attitudes of physicians in low and middle-income countries to those in high-income countries. They should also explore how the healthcare, political and economic structures affect those attitudes. There is also a need to improve the quality of studies in this field, particularly in terms of using validated survey tools. Moreover, a systematic review assessing the knowledge, beliefs and attitudes of patients and the public can assist in planning and implementing policy interventions.

## Conclusions

We identified few studies conducted in low and middle-income countries, each of which had a number of methodological limitations. The top perceived benefits of the interaction between physicians and pharmaceutical companies were receiving information and rewards. While physicians generally perceived the impact of interactions on their behavior to be minor, their attitudes toward receiving information and rewards varied across studies.

## Additional file

Additional file 1:
**Search strategy.** (DOCX 52 kb)

## Abbreviations

PCR: Pharmaceutical company representatives
LMIC: Low and middle-income countries
