# Supplementary material for: Knowledge, beliefs and attitudes of physicians in low and middle-income countries regarding interacting with pharmaceutical companies: a systematic review
Source: BMC Health Serv Res. 2016 Feb 17;16:57. doi: 10.1186/s12913-016-1299-4 (PMC4756506; doi:10.1186/s12913-016-1299-4)
Supplement: Additional file 1: — Search strategy. (DOCX 52 kb) [file 12913_2016_1299_MOESM1_ESM.docx]

**Additional file 1:** Search strategy

**Medline** 1946 to September 2015

1. Conflict of Interest.mp.or "Conflict of Interest"/

2. Drug Industry/

3. Gift Giving/

4. detailman.mp.

5. commercial information.mp.

6. ((drug or pharma*) adj3 (industry or firm* or manufacture* or compan*)).mp.

7. physician*.mp.

8. doctor*.mp.

9. Physicians/

10. primary care.mp.

11. or/1-6

12. or/7-10

13. 11 and 12

14. 13 not (comment or editorial or letter).pt.

**EmBASE** 1980 to April 2014

1. Conflict of Interest.mp.or "Conflict of Interest"/

2. Drug Industry/

3. Gift Giving/

4. detailman.mp.

5. commercial information.mp.

6. ((drug or pharma*) adj3 (industry or firm* or manufacture* or compan*)).mp.

7. physician*.mp.

8. doctor*.mp.

9. Physician/

10. primary care.mp.

11. or/1-6

12. or/7-10

13. 11 and 12

14. 13 not (comment or editorial or letter).pt
